# Supplementary material for: Development and validation of a clinical score for identifying patients with high risk of latent autoimmune adult diabetes (LADA): The LADA primary care-protocol study
Source: PLoS One. 2023 Feb 9;18(2):e0281657. doi: 10.1371/journal.pone.0281657 (PMC9910627; doi:10.1371/journal.pone.0281657)
Supplement: S1 Table — LADA criteria according to the Immunology of Diabetes Society [14]. (DOCX) [file pone.0281657.s001.docx]

**DEVELOPMENT AND VALIDATION OF A CLINICAL SCORE FOR IDENTIFYING PATIENTS WITH HIGH RISK OF LATENT AUTOIMMUNE ADULT DIABETES (LADA): THE LADA PRIMARY CARE-PROTOCOL STUDY.**

**Supporting Information. Tables**

**S1 Table: Main variable. LADA criteria according to the Immunology of Diabetes Society [14].**

| **Criteria** | **Yes** | **No** |
| --- | --- | --- |
| Criterion 1. Adult age of onset of the disease (> 30 years of age) |  |  |
| Criterion 2. Presence of at least one circulating autoantibody against pancreatic islets (GADA / ICA / IAA / IA-2) |  |  |
| Criterion 3. Initial independence of insulin for its control (during the first six months) |  |  |

*LADA Criteria: Yes, if you meet all 3 criteria. No, if you do not meet all 3 criteria.*

*The eDCN has an automatic system that defines the patient as LADA if “Yes” has been marked in the 3 criteria and “No” if some of the 3 criteria are not met.*

*It will be considered that the patient has been independent of insulin for its control during the first 6 months, if it has been adequately controlled with non-insulin drugs for this period. If for a maximum period of 1 month the patient has been treated with insulin during debut of the disease, needing to lower blood glucose quickly, but has subsequently been well controlled with non-insulin drugs, it will be marked that the patient has been insulin independent.*
