# Supplementary material for: Development and validation of a clinical score for identifying patients with high risk of latent autoimmune adult diabetes (LADA): The LADA primary care-protocol study
Source: PLoS One. 2023 Feb 9;18(2):e0281657. doi: 10.1371/journal.pone.0281657 (PMC9910627; doi:10.1371/journal.pone.0281657)
Supplement: S2 Table — (DOCX) [file pone.0281657.s002.docx]

**S2 Table. Sociodemographic variables: age.**

| **Date of birth** | |
| --- | --- |
| Day |  |
| Month |  |
| Year |  |
