# Supplementary material for: Development and validation of a clinical score for identifying patients with high risk of latent autoimmune adult diabetes (LADA): The LADA primary care-protocol study
Source: PLoS One. 2023 Feb 9;18(2):e0281657. doi: 10.1371/journal.pone.0281657 (PMC9910627; doi:10.1371/journal.pone.0281657)
Supplement: S4 Table — (DOCX) [file pone.0281657.s004.docx]

**S4 Table. Sociodemographic variables: Education Level.**

| 1. Cannot read or write |  |
| --- | --- |
| 2. Incomplete elementary school |  |
| 3. Elementary school |  |
| 4. Middle School |  |
| 5. High School |  |
| 6. Vocational Technical School |  |
| 7. Extended Vocational Technical School |  |
| 8. University/College |  |
