# Supplementary material for: Development and validation of a clinical score for identifying patients with high risk of latent autoimmune adult diabetes (LADA): The LADA primary care-protocol study
Source: PLoS One. 2023 Feb 9;18(2):e0281657. doi: 10.1371/journal.pone.0281657 (PMC9910627; doi:10.1371/journal.pone.0281657)
Supplement: S5 Table — (DOCX) [file pone.0281657.s005.docx]

**S5 Table. Sociodemographic variables: Family and Social support.**

| Who takes care of you when you are sick and need help? | |
| --- | --- |
| 1. I have no one to turn to |  |
| 2. My partner |  |
| 3. Another relative who is not my partner |  |
| 4. Another person without family ties (neighbors, friends, etc.) |  |
| 5. A person paid for it |  |
| 6. Social services |  |
| 7. Another situation |  |
