# Supplementary material for: Development and validation of a clinical score for identifying patients with high risk of latent autoimmune adult diabetes (LADA): The LADA primary care-protocol study
Source: PLoS One. 2023 Feb 9;18(2):e0281657. doi: 10.1371/journal.pone.0281657 (PMC9910627; doi:10.1371/journal.pone.0281657)
Supplement: S6 Table — (DOCX) [file pone.0281657.s006.docx]

**S6 Table. Anthropometric variables.**

| Height (cm). |  |
| --- | --- |
| Weight (Kg). |  |
| BMI (Kg / m2) |  |
| Waist circumference (cm) |  |

*Measurement of abdominal waist: Patient standing, with feet together, arms at the sides and abdomen relaxed, then surround his abdomen with the measuring tape at the level of the navel and without pressing, take a deep breath and immediately take the air out. The WHO establishes the maximum healthy value of the abdominal girth at 88 cm in women and 102 cm in men.*
