# Supplementary material for: Development and validation of a clinical score for identifying patients with high risk of latent autoimmune adult diabetes (LADA): The LADA primary care-protocol study
Source: PLoS One. 2023 Feb 9;18(2):e0281657. doi: 10.1371/journal.pone.0281657 (PMC9910627; doi:10.1371/journal.pone.0281657)
Supplement: S8 Table — (DOCX) [file pone.0281657.s008.docx]

**S8 Table.** **Clinical variables: Diagnostic method of DM.**

| 1. Cardinal symptoms (polyuria, polydipsia, weight loss) + blood glucose≥ 200 mg/dl (≥11.1 mmol/L ). |  |
| --- | --- |
| 2. Hb A1c≥ 6.5% (≥48 mmol/mol), on two occasions. |  |
| 3. Two blood glucose figures≥ 126 mg/dl (≥6.99 mmol/L ), on two occasions. |  |
| 4. Blood glucose 2 hours after an oral glucose tolerance test with 75 g of glucose≥ 200 mg/dl (≥11.1 mmol/L ), on two occasions. |  |
| 5. Unknown diagnostic method or performed in another health center. |  |
| 6. Hb A1c≥ 6.5% (≥48 mmol/mol), on at least one occasion + fasting plasma glucose≥ 126 mg/dl (≥6.99 mmol/L ), on another occasion. |  |
