# Supplementary material for: Development and validation of a clinical score for identifying patients with high risk of latent autoimmune adult diabetes (LADA): The LADA primary care-protocol study
Source: PLoS One. 2023 Feb 9;18(2):e0281657. doi: 10.1371/journal.pone.0281657 (PMC9910627; doi:10.1371/journal.pone.0281657)
Supplement: S10 Table — Life habits: Tobacco. (DOCX) [file pone.0281657.s010.docx]

**S10 Table. Clinical variables. Life habits: Tobacco.**

| 1. Active smoker (person who currently smokes, either daily or occasionally) |  | Number of cigarettes / day (currently or in the past): Numeric value |  |
| --- | --- | --- | --- |
| 2. Former smoker (person who have not smoked for at least 1 year) |  | Number of years smoker: Numerical value |  |
|  |  | Number of packages per year |  |
| 3. Has never smoked |  | | |

*Note: The eDCN incorporates a calculator that automatically calculates the number of packages per year*
