# Supplementary material for: Development and validation of a clinical score for identifying patients with high risk of latent autoimmune adult diabetes (LADA): The LADA primary care-protocol study
Source: PLoS One. 2023 Feb 9;18(2):e0281657. doi: 10.1371/journal.pone.0281657 (PMC9910627; doi:10.1371/journal.pone.0281657)
Supplement: S11 Table — Life habits: Alcohol. (DOCX) [file pone.0281657.s011.docx]

**S11 Table. Clinical variables. Life habits: Alcohol.**

| **Type of alcoholic drink** | **Daily alcohol units** | **Weekly alcohol units** |
| --- | --- | --- |
| **Beer** |  |  |
| **Wine** |  |  |
| **Vermouth** |  |  |
| **Spirits** |  |  |

*The daily and weekly units of alcohol will be recorded by adding the consumption of units. One unit (10 g of alcohol) is equivalent to: a glass of wine (100 cc), half of a pint of beer (200 cc), half a glass of vermouth (50 cc) or half a glass of whiskey or cognac (25 cc).*

*Volume measurements in ml or cc are: small cup, 125 ml. Tube/small glass, 200 ml. Large glass, 250 ml. Cup/ Combined, 50 ml. Bottle, 200 ml. Wine bottle: 750 ml.*

*The eDCN includes a calculator that allows calculating daily and weekly alcohol consumption and considering whether the patient is a risk drinker (> 40 g of alcohol / day in men or > 24 g of alcohol/day in women)*
