# Supplementary material for: Development and validation of a clinical score for identifying patients with high risk of latent autoimmune adult diabetes (LADA): The LADA primary care-protocol study
Source: PLoS One. 2023 Feb 9;18(2):e0281657. doi: 10.1371/journal.pone.0281657 (PMC9910627; doi:10.1371/journal.pone.0281657)
Supplement: S12 Table — Life habits: Physical activity level [37]. (DOCX) [file pone.0281657.s012.docx]

**S12 Table. Clinical variables. Life habits:** **Physical activity level [37].**

| Low physical activity |  |
| --- | --- |
| Moderate physical activity |  |
| High physical activity |  |

*Definitions of Physical activity level are included in the eDCN.*
