# Supplementary material for: Development and validation of a clinical score for identifying patients with high risk of latent autoimmune adult diabetes (LADA): The LADA primary care-protocol study
Source: PLoS One. 2023 Feb 9;18(2):e0281657. doi: 10.1371/journal.pone.0281657 (PMC9910627; doi:10.1371/journal.pone.0281657)
Supplement: S13 Table — Life habits: Physical involvement work [37]. (DOCX) [file pone.0281657.s013.docx]

**S13 Table. Clinical variables. Life habits: Physical involvement work** **[37].**

| The patient has a job that involves physical activity |  |
| --- | --- |
| The patient has a sedentary job |  |

*Definitions are included in the eDCN.*
