# Supplementary material for: Development and validation of a clinical score for identifying patients with high risk of latent autoimmune adult diabetes (LADA): The LADA primary care-protocol study
Source: PLoS One. 2023 Feb 9;18(2):e0281657. doi: 10.1371/journal.pone.0281657 (PMC9910627; doi:10.1371/journal.pone.0281657)
Supplement: S14 Table — Life habits: Mediterranean diet [38]. (DOCX) [file pone.0281657.s014.docx]

**S14 Table. Clinical variables. Life habits: Mediterranean diet** [38].

| Answer the questions | Yes | No |
| --- | --- | --- |
| 1. Do you use olive oil as your main culinary fat? |  |  |
| 1. How much olive oil do you consume in a day? (including oil used for frying, salads, meals from home, etc.) More than 3 tablespoons? |  |  |
| 1. How many servings of raw vegetables or salad do you consume per day? (1 serving = 200g [consider side dishes as half a serving]) More than 1? |  |  |
| 1. How many units of fruit (including natural fruit juices) do you consume per day? More than 2? |  |  |
| 1. How many servings of red meat, hamburger, or meat products (ham, sausage, etc.) do you consume per day? |  |  |
| 1. How many servings of butter, margarine or cream do you consume per day? (one serving = 12g) Less than 1? |  |  |
| 1. How many sweet or carbonated drinks do you drink per day? Less than 1? |  |  |
| 1. How much wine do you drink per week? More than 2 glasses? |  |  |
| 1. How many servings of legumes do you eat per week? (1 serving = 150 g) More than 2? |  |  |
| 1. How many servings of fish or shellfish do you eat per week? (1 portion = 100-150 g of fish or 4-5 units or 200 g of shellfish) More than 2? |  |  |
| 1. How many times a week do you consume candy or cakes (not homemade), such as cakes, cookies, biscuits or custard? Less than 3? |  |  |
| 1. How many servings of nuts (including peanuts) do you consume per week? (One serving = 30 g) 1 or more than 1? |  |  |
| 1. Do you prefer chicken, turkey or rabbit meat instead of beef, pork, hamburgers or sausages? |  |  |
| 1. Do you consume vegetables, pasta, rice, or other dishes seasoned with “sofrito” more than once a week? (sauce made with tomato and onion, leek or garlic and simmered with olive oil) |  |  |

*The diet is considered to be Mediterranean if the score is between 11 and 14 points*
