# Supplementary material for: Development and validation of a clinical score for identifying patients with high risk of latent autoimmune adult diabetes (LADA): The LADA primary care-protocol study
Source: PLoS One. 2023 Feb 9;18(2):e0281657. doi: 10.1371/journal.pone.0281657 (PMC9910627; doi:10.1371/journal.pone.0281657)
Supplement: S15 Table — (DOCX) [file pone.0281657.s015.docx]

**S15 Table. Laboratory parameters.**

| Blood glucose value at diagnosis (mg/dl and mmol/L) |  | | | | |
| --- | --- | --- | --- | --- | --- |
| Hb A1c value at diagnosis: Numerical value (% and mmol/mol) |  | | | | |
| HbA1c value in the last 6 months: Numerical value (% and mmol/mol) |  | | | | |
| Albumin / creatinine ratio: Numerical value |  | | | | |
| Has the patient ever had ketonuria? | Yes | No | | Unknow | |
|  |  |  | |  | |
| Total cholesterol: Numerical value (mg/dl) |  | | | | |
| HDL cholesterol: Numerical value (mg/dl) |  | | | | |
| LDL cholesterol: Numerical value (mg/dl) |  | | | | |
| Triglycerides: Numerical value (mg/dl) |  | | | | |
| Anti GAD 65 antibodies: (+/- and titer -IU/mL-) | Positive | | Titer | | Negative |
|  |  | |  | |  |
|  |  | |  | |  |
| Anti-peroxidase antibodies: (+/- and titer-IU/mL-) | Positive | | Titer | | Negative |
|  |  | |  | |  |
|  |  | |  | |  |
| Antithyroglobulin antibodies: (+/- and titer-IU / mL-) | Positive | | Titer | | Negative |
|  |  | |  | |  |
|  |  | |  | |  |
