# Supplementary material for: Development and validation of a clinical score for identifying patients with high risk of latent autoimmune adult diabetes (LADA): The LADA primary care-protocol study
Source: PLoS One. 2023 Feb 9;18(2):e0281657. doi: 10.1371/journal.pone.0281657 (PMC9910627; doi:10.1371/journal.pone.0281657)
Supplement: S16 Table — (DOCX) [file pone.0281657.s016.docx]

**S16 Table. Treatment for diabetes mellitus.**

|  | | Yes, currently | Yes, but not currently | Time, in months | Never |
| --- | --- | --- | --- | --- | --- |
| Lifestyle actions | |  |  |  |  |
| Metformin | |  |  |  |  |
| Sulfonylureas | Glibenclamide |  |  |  |  |
|  | Gliclazide |  |  |  |  |
|  | Glimepiride |  |  |  |  |
|  | Glipizide |  |  |  |  |
|  | Glisentide |  |  |  |  |
|  | | | | | |
| Dipeptydil-peptidase-4 inhibitors | Sitagliptin |  |  |  |  |
|  | Vildagliptin |  |  |  |  |
|  | Linagliptin |  |  |  |  |
|  | Saxagliptin |  |  |  |  |
|  | Alogliptin |  |  |  |  |
|  | | | | | |
| Glitazonas (Pioglitazone) | |  |  |  |  |
|  | | | | | |
| Glinidas (Repaglinide) | |  |  |  |  |
|  | | | | | |
| SGLT-2 inhibitors | Canagliflozin |  |  |  |  |
|  | Empagliflozin |  |  |  |  |
|  | Dapagliflozin |  |  |  |  |
|  | Ertugliflozin |  |  |  |  |
|  | | | | | |
| GLP-1 analogs | Exenatide |  |  |  |  |
|  | Liraglutide |  |  |  |  |
|  | Semaglutide, |  |  |  |  |
|  | Lixisenatide |  |  |  |  |
|  | Albiglutide |  |  |  |  |
|  | Dulaglutide |  |  |  |  |
|  | | | | | |
| Insulin | |  |  |  |  |
| Date of initiation of insulin treatment (dd/mm/yyyy). | |  | | | |
| End date of insulin treatment (dd/mm/ yyyy). | |  | | | |

*Action on lifestyles (if it appears in the EHR, or the patient refers that lifestyles have been indicated in relation to his pathology)*
