# Supplementary material for: Development and validation of a clinical score for identifying patients with high risk of latent autoimmune adult diabetes (LADA): The LADA primary care-protocol study
Source: PLoS One. 2023 Feb 9;18(2):e0281657. doi: 10.1371/journal.pone.0281657 (PMC9910627; doi:10.1371/journal.pone.0281657)
Supplement: S17 Table — (DOCX) [file pone.0281657.s017.docx]

**S17 Table. Other treatments.**

| Drug | Yes, currently | Yes, but not currently | Time, in months |
| --- | --- | --- | --- |
| Angiotensin converting enzyme inhibitors (ACEI)/ angiotensin receptor blockers (ARBs) |  |  |  |
| Non-ACEI / ARBs antihypertensive drugs |  |  |  |
| Lipid-lowering drugs |  |  |  |
| Anxiolytics and hypnotics drugs |  |  |  |
| Antidepressants drugs |  |  |  |
| Antipsychotics drugs |  |  |  |
| Antiplatelet drugs |  |  |  |
| Anticoagulant drugs |  |  |  |
| Analgesics |  |  |  |
| Anti-tumor drugs |  |  |  |
| Immunomodulator drugs |  |  |  |
| Corticosteroids |  |  |  |

*Angiotensin converting enzyme inhibitors (ACEI)/ angiotensin receptor blockers (ARBs): ACEI: Benazepril, Captopril, Enalapril, Fosinopril, Lisinopril, Perindopril, Quinapril, Ramipril, Trandolapril, Candesartan, Eprosartan, Irbesartan, Losartan, Olmesartan, Telemisartan, Valsartan among the most common.*

*Non-ACEI / ARBs antihypertensive drugs: Diltiazem, Verapamil, Nifedipine, Amlodipine, Lercanidipine, Manidipine, Felodipine, Doxazosin, among the most common.*

*Lipid-lowering drugs: Atorvastatin, Pitavastatin, Pravastatin, Rosuvastatin, Simvastatin, Ezetimibe, Fenofibrate, Gemfibrozil, Resincolestyramine, among the most common.*

*Anxiolytics and hypnotic drugs: Diazepam, Lorazepam, Lormetazepam, Chlorazepatodipotassium, Bromazepam, Clonazepam, Midazolam, Oxazepam, Alprazolam, Zopliclone, Zolpidem, Trazodone (at low doses at night, up to 100 mg / night) and Mirtazapine at low doses at night, up to 15 mg / night), among the most common.*

*Antidepressant drugs: Fluoxetine, Setralin, Citalopram, Escitalopram, Fluvoxamine, Venlafaxine, Desvenlafaxine, Duloxetine, Bupropion, Vortioxetine, Agomelatine, Trazodone at high doses (greater than 100 mg / day), Mirtazapine (doses greater than 15 mg / day, among the most common.*

*Antipsychotic drugs: Haloperidol, Risperidone, Quetiapine, Clozapine, Olanzapine, Aripiprazole, Ziprasidone among the most* *common.*

*Antiplatelet drugs: Acetylsalicylic acid, Clopidogrel, Ticagrelol, among the most common.*

*Anticoagulant drugs: Acenocoumarol, Rivaroxaban, Apixaban, Dabigatran, Edoxaban.*

*Analgesic drugs: Paracetamol, Metamizole, Tramadol, Ibuprofen, Naproxen, Diclofenac, Dexketoprofen, Etoricoxib, Celecoxib, Codeine, Morphine, Fentanyl, Tapentadol, among the most common.*

*Anti-tumor drugs: Any treatment that the patient reports that has been prescribed for cancer or that appears in the patient's history*

*Immunomodulator drugs, including biological agents (generally used for chronic inflammatory and rheumatic diseases, some types of cancer, or multiple sclerosis): Methotrexate, Leflunomide, Hydroxychloroquine, Chloroquine, Azathioprine, Cyclosporin A, Cyclophosphamide, Tocilizumab, Etanercept, Infliximab, Anakinbidumab, Anakinbrailid Abatacept, Rituximab, Golimumab, Canakinumab, Ustekinumab, Denosumab, Secukinumab, Belibumab, Certolizumab, among the most common.*

*Corticosteroids: Hydrocortisone, Prednisone, Prednisolone, Deflazacort, Dexamethasone.*
