# Supplementary material for: Development and validation of a clinical score for identifying patients with high risk of latent autoimmune adult diabetes (LADA): The LADA primary care-protocol study
Source: PLoS One. 2023 Feb 9;18(2):e0281657. doi: 10.1371/journal.pone.0281657 (PMC9910627; doi:10.1371/journal.pone.0281657)
Supplement: S18 Table — (DOCX) [file pone.0281657.s018.docx]

**S18 Table. Comorbidities**.

| Disease | Yes | Date (year) | No | Unknown |
| --- | --- | --- | --- | --- |
| Microalbuminuria |  |  |  |  |
| Chronic Kidney Disease |  |  |  |  |
| Diabetic retinopathy |  |  |  |  |
| Diabetic neuropathy |  |  |  |  |
| High blood pressure |  |  |  |  |
| Hypercholesterolemia |  |  |  |  |
| Hypertriglyceridemia |  |  |  |  |
| Ischemic heart disease |  |  |  |  |
| Peripheral arterial disease |  |  |  |  |
| Cerebrovascular disease |  |  |  |  |
| Atrial fibrillation |  |  |  |  |
| Heart failure |  |  |  |  |
| COPD |  |  |  |  |
| Asthma |  |  |  |  |
| Mood disorders: anxiety, depression, dysthymia, insomnia or prescription of chronic drugs for these indications |  |  |  |  |
| Psychotic disorder |  |  |  |  |
| Cancer |  |  |  |  |

*The patient is considered to have a disease if it is registered in the medical record or the patient can prove it*
