# Supplementary material for: Development and validation of a clinical score for identifying patients with high risk of latent autoimmune adult diabetes (LADA): The LADA primary care-protocol study
Source: PLoS One. 2023 Feb 9;18(2):e0281657. doi: 10.1371/journal.pone.0281657 (PMC9910627; doi:10.1371/journal.pone.0281657)
Supplement: S19 Table — (DOCX) [file pone.0281657.s019.docx]

**S19 Table. Metabolic Syndrome.**

| Criteria | Yes | No |
| --- | --- | --- |
| Abdominal circumference≥ 102 cm in men and ≥88 cm in women |  |  |
| Triglycerides ≥ 150 mg/dl |  |  |
| Blood pressure ≥ 130/85 mm / Hg or established arterial hypertension |  |  |
| HDL Cholesterol < 40 mg/dl in men and <50 mg/dl in women |  |  |
| Fasting plasma glucose 110 to 126 mg/dl (6.11 to 6.99 mmol/L ) or DM |  |  |

*At least 3 of the following criteria (The eDCN incorporates a calculator that automatically marks “YES” when at least 3 criteria are met and “No” when less than 3 are met*
