# Supplementary material for: Development and validation of a clinical score for identifying patients with high risk of latent autoimmune adult diabetes (LADA): The LADA primary care-protocol study
Source: PLoS One. 2023 Feb 9;18(2):e0281657. doi: 10.1371/journal.pone.0281657 (PMC9910627; doi:10.1371/journal.pone.0281657)
Supplement: S20 Table — (DOCX) [file pone.0281657.s020.docx]

**S20 Table. Personal history of autoimmune disorder linked to HLA DR3 / DQ2 or DR4 / DQ8.**

| Disease | Yes | No | Unknow |
| --- | --- | --- | --- |
| Autoimmune thyroid disease |  |  |  |
| Pernicious anemia |  |  |  |
| Celiac disease |  |  |  |
| Addison's disease |  |  |  |
| Vitiligo |  |  |  |
| Rheumatoid arthritis |  |  |  |
| Autoimmune hepatitis |  |  |  |

*The patient will be considered to have autoimmune thyroid disease if it is recorded in the EHR, or the patient document having an alteration in thyroid function associated with the presence of antithyroid antibodies. Also, if the diagnoses of hypothyroidism, hyperthyroidism, or antithyroid or thyroxine treatments are documented, and in addition, the patient has positive antithyroid antibodies. (The requests by the researcher as part of the study are valid). The patient will not be considered to have autoimmune thyroid disease if he only has positive anti-thyroid Ab. For the rest of diseases, they must appear in the clinical records, or the patient must prove it by means of a medical document.*
