# Supplementary material for: Development and validation of a clinical score for identifying patients with high risk of latent autoimmune adult diabetes (LADA): The LADA primary care-protocol study
Source: PLoS One. 2023 Feb 9;18(2):e0281657. doi: 10.1371/journal.pone.0281657 (PMC9910627; doi:10.1371/journal.pone.0281657)
Supplement: S21 Table — (DOCX) [file pone.0281657.s021.docx]

**S21 Table. First or second-degree family history (parents, children, siblings, grandparents, great-uncles, and nephews) of an autoimmune disorder linked to HLA DR3 / DQ2 or DR4 / DQ8.**

| Disease | Yes | No | Unknown | Number of affected relatives |
| --- | --- | --- | --- | --- |
| Autoimmune thyroid disease |  |  |  |  |
| Pernicious anemia |  |  |  |  |
| Celiac disease |  |  |  |  |
| Addison's disease |  |  |  |  |
| Vitiligo |  |  |  |  |
| Rheumatoid arthritis |  |  |  |  |
| Autoimmune hepatitis |  |  |  |  |
| T1DM |  |  |  |  |

*For the Family History of these diseases, the patient may prove, or simply refer, the existence of these pathologies.*
