# Supplementary material for: Development and validation of a clinical score for identifying patients with high risk of latent autoimmune adult diabetes (LADA): The LADA primary care-protocol study
Source: PLoS One. 2023 Feb 9;18(2):e0281657. doi: 10.1371/journal.pone.0281657 (PMC9910627; doi:10.1371/journal.pone.0281657)
Supplement: S22 Table — (DOCX) [file pone.0281657.s022.docx]

**S22 Table. Perceived health status: Excellent / Very good / Good / Fair / Poor.**

| Perceived health status | Excellent | Very good | Good | Fair | Poor |
| --- | --- | --- | --- | --- | --- |
|  |  |  |  |  |  |
