# Supplementary material for: Development and validation of a clinical score for identifying patients with high risk of latent autoimmune adult diabetes (LADA): The LADA primary care-protocol study
Source: PLoS One. 2023 Feb 9;18(2):e0281657. doi: 10.1371/journal.pone.0281657 (PMC9910627; doi:10.1371/journal.pone.0281657)
Supplement: S23 Table — (DOCX) [file pone.0281657.s023.docx]

**S23 Table. Fourlanos criteria (LADA Clinical Score by Fourlanos et al** [35]**.**

| Criteria | Yes | No |
| --- | --- | --- |
| Age <50 years at diagnosis of DM |  |  |
| Body Mass Index at diagnosis <25 Kg/m^2^ |  |  |
| Symptoms of hyperglycemia at diagnosis (*) |  |  |
| Personal history of autoimmune disease (**) |  |  |
| Family history of autoimmune disease (**) |  |  |

*The patient will be considered to meet the Fourlanos criteria if they have 2 or more criteria, and not to meet them if they have 1 or no criteria. The eDCN will automatically calculate whether the patient meets the criteria or not, based on the number of positive criteria.*

*(*) Polyuria, polydipsia and weight loss not attributable to another condition*

*(**) Autoimmune disorders linked to HLA DR3 / DQ2 or DR4 / DQ8: autoimmune thyroid disease, pernicious anemia, celiac disease, Addison's disease, vitiligo, rheumatoid arthritis, autoimmune hepatitis, T1DM*
